# Mitigation Effect of Proanthocyanidin on Secondary Heart Injury in Rats Caused by Mechanical Trauma

**DOI:** 10.1038/srep44623

**Published:** 2017-03-15

**Authors:** Shuo Ma, Chong Chen, Tingting Cao, Yue Bi, Jicheng Zhou, Xintao Li, Deqin Yu, Shuzhuang Li

**Affiliations:** 1 Dalian Medical University, Department of Physiology, Dalian, 116044, China

## Abstract

Multiple organ dysfunctional syndrome secondary to mechanical trauma (MT) has attracted considerable research attention. The heart is one of the most important organs of the body, and secondary cardiac insufficiency caused by MT seriously affects the quality of life. This study aims to investigate whether proanthocyanidin can alleviate myocardial injury and improve heart function in the process of MT leading to secondary cardiac insufficiency. Noble-Collip drum wasused to prepare MT model in rats. And myocardial apoptosis index was calculated after TUNEL staining. Ventricular intubation was employed to detect heart function. Changes in myocardial ultrastructure were observed using an electron microscope. ELISA was used to detect the content of TNF-α and reactive oxygen species generated from monocytes and cardiomyocytes. The changes in Ca^2+^ concentration in cardiomyocyte were observed by confocal microscope. Compared with trauma group, the administration group had a decreased apoptosis index of cardiomyocytes, and increased ±dp/dtmax. Meanwhile, proanthocyanidin can inhibit monocytes’ TNF-α production, and reduce plasma TNF-α concentration. Moreover, proanthocyanidin can attenuate the excessive oxidative stress reaction of cardiomyocyte, and inhibit calcium overload in cardiomyocytes. In conclusion, proanthocyanidin can effectively ease myocardial damage and improve cardiac function, through anti-inflammatory and antioxidant effects in secondary cardiac insufficiency caused by MT.

Mechanical trauma (MT) is the organ dysfunction or structure damage subjected to mechanical violence. There are many causes of MT, blunt and penetrating, including falls, motor vehicle collisions, and gunshot wounds. Multiple organ dysfunctional syndrome (MODS) secondary to MT has been given particular attention in current medical research of trauma^1^,^2^,^3^. After blunt chest trauma and crush injury, some patients do not have coronary artery transection injury, cardiac contusion, and primary cardiac injury during early clinical observation period, but present secondary cardiac insufficiency in the later period of trauma^4^,^5^. The heart is one of the most important organs of the body and secondary cardiac insufficiency caused by MT seriously affects quality of life^4^,^6^,^7^.

Studies showed that significant myocardial cell apoptosis can be found 6 hours after trauma, reaching the peak in 12 hours. Then, cardiac dysfunction occurs in rats. Thus, myocardial cell apoptosis in rats caused by MT is an important pathological basis of secondary cardiac insufficiency. Meanwhile, a significant rise of reactive oxygen free radicals (ROS) in plasma after MT in rats is an important cause of myocardial cell apoptosis in rats^8^,^9^,^10^. Our previous experiments proved that, the upstream mediators resulting in myocardial cell apoptosis after MT exists in plasma, mainly from the injured tissue and blood, but not from the heart muscle. Pharmacological and gene knockdown analyses showed that the pathological medium is mainly TNF-α. Triggering the overexpression of iNOS/NADPH oxidase can increase NO and superoxide anion production, followed by an increase of ONOO^−^ formation, and the enhancement of caspase-3 activity, thereby causing myocardial cell apoptosis^10^,^11^. Therefore, during the pathological process of secondary cardiac insufficiency caused by MT, drugs that can effectively inhibit the release of the inflammatory mediator TNF-α and the production of superoxide anion are desired for effectively reducing myocardial cell apoptosis and improving heart function, and thus improving the survival rate of patients with MT.

Proanthocyanidin (PC) is a natural polyphenolic compound with antioxidant, anti-inflammatory, and antitumor effects, which widely exist in plants, especially grape seed. Grape seed proanthocyanidin extract (GSPE) has been widely studied because it contains high amount of phenolic hydroxyl groups, which endow its antioxidant and free-radical scavenging capacity^12^,^13^,^14^,^15^. Moreover, GSPE is a natural, effective antioxidant, with good water solubility and high bioavailability, and can be absorbed by the body. With the continuous improvement of purification methods^16^, GSPE has a good prospect for practical application in terms of guaranteed activity. This study aims to investigate whether proanthocyanidin extracted from grape seeds can effectively alleviate myocardial injury and improve heart function through its anti-inflammatory and antioxidative effects in the pathological process of MT leading to secondary myocardial cell apoptosis in rats, and provide a theoretical basis for developing proanthocyanidin as a drug for preventing secondary cardiac insufficiency caused by MT and MODS secondary to trauma.

## Results

### GSPE effectively inhibit myocardial cell apoptosis in rats caused by MT

**Figure 1:**
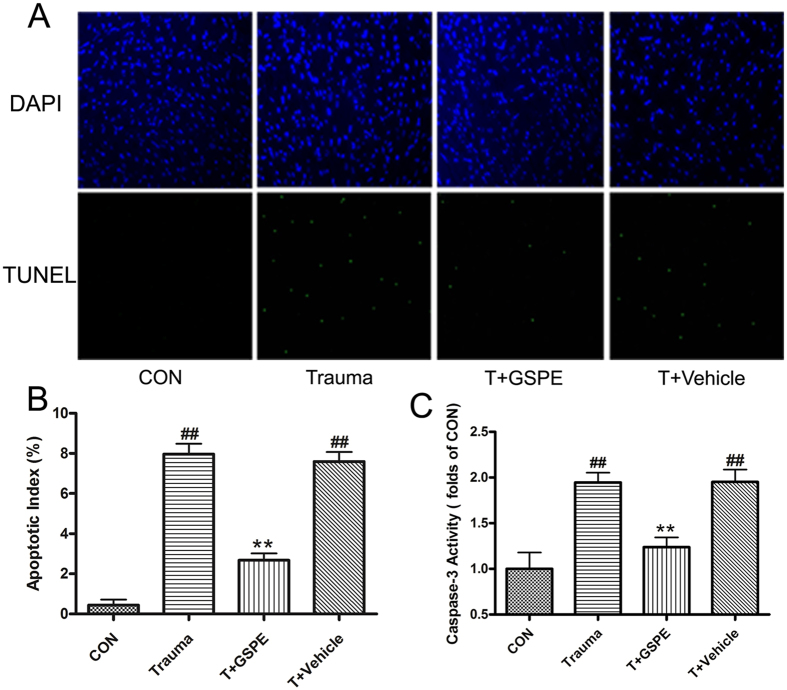
GSPE can effectively inhibit myocardial cell apoptosis in rats caused by MT. GSPE attenuated trauma induced cardiomyocyte apoptosis determined by TUNEL staining (**A**,**B**) and caspase-3 activation (**C**). Cardiomyocytes’ total nuclei were determined by DAPI staining (blue), and apoptotic nuclei were identified by positive TUNEL staining (green) were counted using an image analysis program and the apoptotic index was automatically calculated (number of positively stained myocytes/total number of myocytes × 100%); Caspase-3 activity was detected by a fluorescent kit and normalized against the mean value of the trauma group. n = 5 rats in each group. ^##^P < 0.01 vs CON group, **P < 0.01 vs T group.

In 12 hours after 200r-strength MT, myocardial tissues were obtained after rat was anesthetized, and sample slices were prepared. TUNEL staining was employed for comparing the difference in the number of apoptosis myocardial cells among groups. The result showed that the number of apoptosis myocardial cells in administration group was significantly lower than that in the trauma group. The corresponding apoptosis coefficient was calculated (2.68 ± 0.35 vs. 7.96 ± 0.52, P < 0.01) and caspase-3 content quantitative analysis index was determined (1.24 ± 0.11 vs. 1.95 ± 0.11, P < 0.01). These indexes were obviously lower than in the administration group than those in the trauma group. Thus, GSPE can effectively inhibit myocardial cell apoptosis 12 hours after non-lethal MT in rats (Fig. 1).

### GSPE effectively improve cardiac insufficiency in rat caused by MT

**Figure 2:**
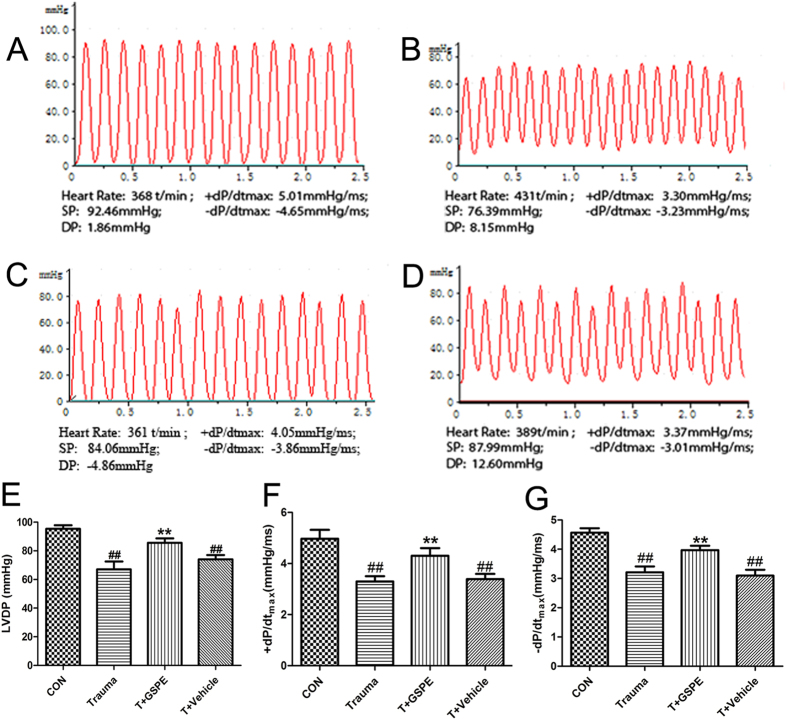
GSPE can effectively improve cardiac insufficiency in rat caused by MT. The typical left ventricular cavity pressure tracing of control group (**A**), Trauma group (**B**), Trauma+ GSPE group (**C**), Trauma+ Vehicle group (**D**). Left ventricular developed pressure (LVDP) (**E**), +dP/dt_max_ (**F**), and −dP/dt_max_ (**G**) were measured in traumatic rats with treatment of GSPE (12 h after 200 revolutions’ trauma). n = 5 rats in each group. ^##^P < 0.01 vs CON group, **P < 0.01 vs Trauma group.

Left ventricular intubation was applied 12 hours after 200r-strength MT in rats. The changes in left ventricular pressure waveforms were recorded, and the LVDP, +dp/dtmax and −dp/dtmax index, which reflect ventricular diastolic function, were calculated. The results showed that the LVDP (85.67 ± 1.77 vs. 67.00 ± 3.22 mmHg, P < 0.01), +dp/dtmax (4.30 ± 0.17 vs. 3.30 ± 0.12 mmHg/ms, P < 0.01) and −dp/dtmax (3.97 ± 0.09 vs. 3.21 ± 0.12 mmHg/ms, P < 0.01) in the administration group were significantly increased compared with the trauma group, and the heart function of rat was significantly improved (Fig. 2).

### GSPE improve the changes in rat myocardial ultrastructure caused by MT

**Figure 3:**
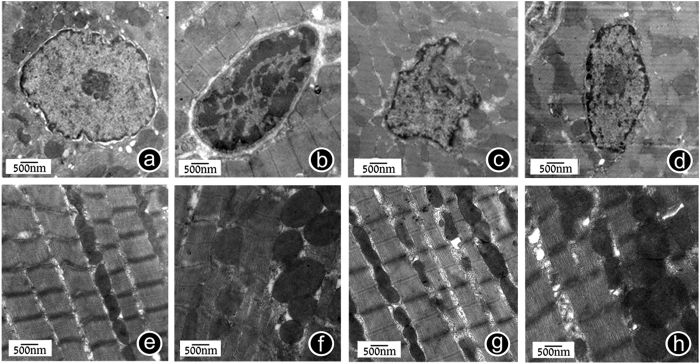
GSPE can improve the changes in rat myocardial ultrastructure caused by MT. (**a**) Normal myocardial cell’s nucleus for reference (15000x). Finely granular appearance of chromatin is present. (**b**) Trauma group exhibited condensed chromatin in part of nucleus and distributed in the edge of nuclear membrane in blocks (15000x). (**c**) After drug interference, chromatin condensation of rat myocardial cells was obviously eased, and nuclear morphology had recovered (15000x). (**d**) Trauma+Vehicle group The nuclear morphology was still abnormal, the nuclear membrane changed in continuity, and the nuclear membrane invaginated and wrinkled (15000x). (**e**) Normal myocardial cell’s mitochondria for reference (15000x). (**f**) Myocardial cell’s mitochondria in the trauma group were swollen, and the individual parts of mitochondria were dissolved, showing vacuole formation (15000x). (**g**) After GSPE interference, mitochondrial swelling was eased and the number of formed vacuoles was reduced (15000x). (**h**) Trauma+Vehicle group (15000x). n = 3 rats in each group.

In 12 hours after 200r-strength MT, myocardial tissues were obtained after rat was anesthetized. After treatment, a transmission electron microscope was used for observation. Compared with the normal group, electron microscope analyses showed that the rat myocardial ultrastructure in the trauma group exhibited condensed chromatin in part of nucleus and distributed in the edge of nuclear membrane in blocks; in addition, the nuclear morphology was abnormal, the nuclear membrane changed in continuity, and the nuclear membrane invaginated and wrinkled. After drug interference, chromatin condensation of rat myocardial cells was obviously eased, and nuclear morphology had recovered. Moreover, compared with the normal group, myocardial cell’s mitochondria in the trauma group were swollen with disrupted crest, and the individual parts of mitochondria were dissolved, showing vacuole formation. After GSPE interference, mitochondrial swelling was eased and the number of formed vacuoles was reduced (Fig. 3).

### GSPE reduced circulating TNF-α level

In our previous study, cardiomyocytes were cultivated with cytomix (a mixture of IFN-γ, IL-1β, and TNF-α) and with IFN-γ, IL-1β, or TNF-α alone. The caspase-3 activation of cardiomyocytes incubated with cytomix and TNF-α was much higher than that with normal plasma. In addition, the exposure of normal cardiomyocytes to TP revealed apparent cell apoptosis; this phenomenon was virtually abolished through the incubation of TP with anti-TNF-α or TP isolated from TNF-α−/− mice, indicating that TNF-α plays a critical role in cardiac injury after MT^11^.

**Figure 4:**
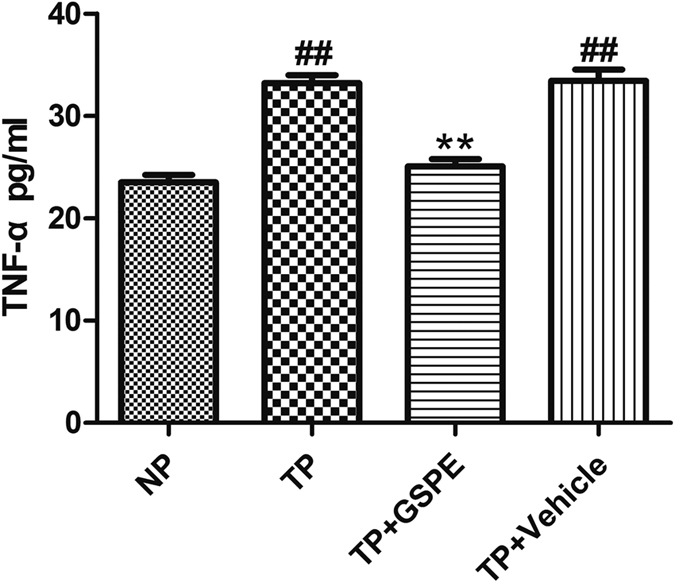
Plasma TNF-α concentration induced by trauma can be attenuated by GSPE. The figure shows the serum concentration of TNF-α 1.5 h after trauma, which was significantly attenuated by pretreatment with GSPE (40 mg/kg i.p. 0.5 h before trauma). n = 3~5 rats in each group. ^##^P < 0.01 vs NP group, **P < 0.01 vs TP group.

One and a half hours after non-lethal MT, aortic blood of rats was obtained to extract plasma and detect the content of TNF-α. ELISA kits were used to determine the amount of TNF-α in the circulatory system 1.5 hours after MT in the four groups. Compared with the trauma group, the content of TNF-α in rat plasma in the administration group had decreased (25.08 ± 0.71 vs. 33.22 ± 0.78 pg/ml, P < 0.01) (Fig. 4). With GSPE pretreatment, overproduction of TNF-α was significantly inhibited, whereas there was no apparent change in TNF-α in the Trauma+Vehicle group compared with the trauma group.

### Suitable concentration of GSPE for H9c2 cells

**Figure 5:**
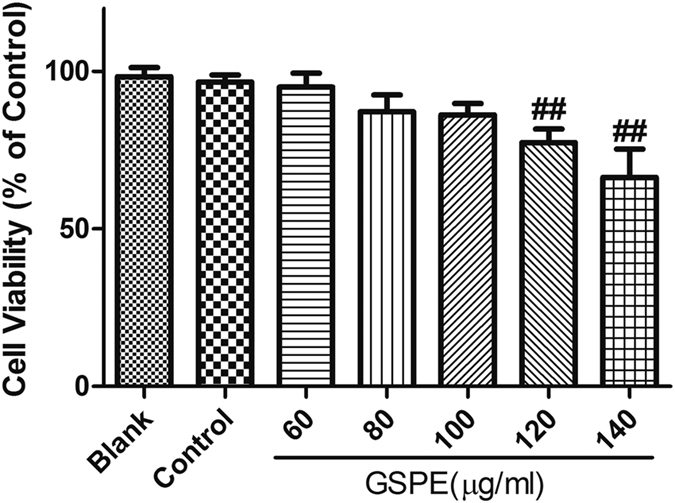
The viability of H9c2 cells was influenced by GSPE concentration. Cells were incubated for 24 h in a 96-well microplate with various concentrations of GSPE (60, 80, 100, 120 and 140 μg/ml). The cytotoxicity of GSPE was evaluated using MTT assay kit, and expressed as a percentage of viable cells comparing to the control group. GSPE showed no cytotoxicity at the concentrations of 60 μg/ml, 80 μg/ml as well as 100 μg/ml, which decreased significantly at the concentrations of 120 μg/ml and 140 μg/ml. n = 3 per group, ^##^P < 0.01 vs. Control group.

The aim of the present study was to gain a better understanding of the anti-inflammatory and antioxidant effects of GSPE in secondary cardiac insufficiency caused by MT. Firstly, MTT assay was performed to determine the appropriate concentration of GSPE in H9c2 cells for *in vitro* test. Cells were treated with GSPE at concentrations of 60, 80, 100, 120, or 140 μg/ml for 24 hours. As shown in Fig. 5, no significant difference in viability was observed at the GSPE concentrations of 60, 80 and 100 μg/ml. The significant cytotoxic effect of GSPE was observed at the concentration of 120 μg/ml. Thus, concentrations under 100 μg/ml would be safe for H9c2 cells.

### GSPE suppressed ROS overproduction in H9c2 cells

**Figure 6:**
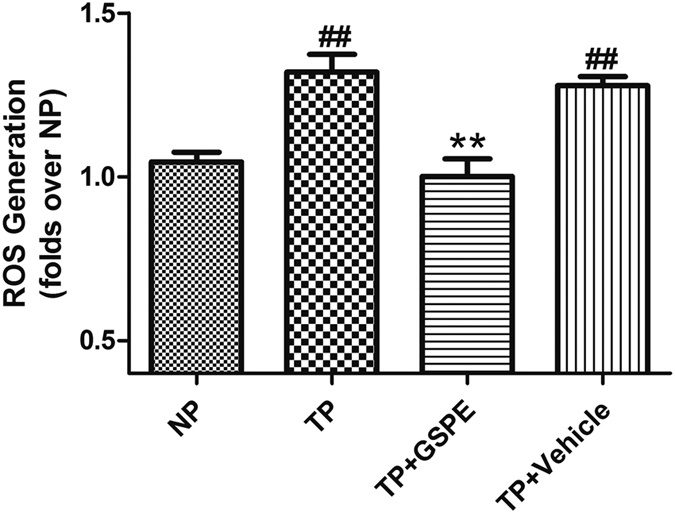
Trauma Induced myocardial cells’ ROS overproduction can be effectively attenuated by GSPE. Intracellular ROS production was determined using DCFH-DA. H9c2 cell oxidative stress was initiated by TP, and was significantly attenuated by pretreatment with GSPE (100 μg/ml), ^##^P < 0.01 vs NP group, **P < 0.01 vs TP group.

Oxidative stress was proven in our previous study to play an important role in MT-induced cardiomyocyte apoptosis. Therefore, we examined the protective effect of GSPE on TP-induced oxidative imbalance in H9c2 cells. Trauma plasma (50% volume fraction) was used to stimulate H9c2 myocardial cells. Rat myocardial cell model by MT in inflammatory mediators was constructed through stimulation to further detect ROS content of myocardial cells. The ROS content in the trauma group was significantly higher than that in the control group (1.32 ± 0.05 vs. 1.05 ± 0.03, P < 0.01). The ROS content in the drug interference group was significantly lower than that in the trauma plasma (1.00 ± 0.05 vs. 1.32 ± 0.05, P < 0.01), close to that in the control group (1.00 ± 0.05 vs. 1.05 ± 0.03, P > 0.05) (Fig. 6).

### GSPE attenuated LPS-induced monocytes’ TNF-α overproduction

**Figure 7:**
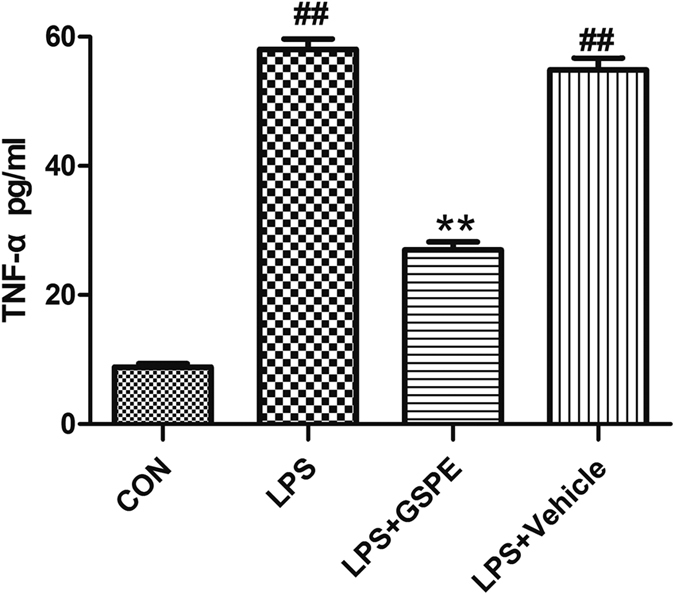
LPS induced monocytes’ TNF-α overproduction can be effectively attenuated by GSPE. TNF-α concentration of THP-1 cells incubated with LPS (1 μg/ml) for 12 h. ^##^P < 0.01 vs CON group, **P < 0.01 vs LPS group.

Lipopolysaccharide (LPS), as a powerful inflammation factor, can activate monocyte macrophage, and enhance the expression of TNF-α gene, causing the abnormal increase of plasma TNF-α. After stimulation of THP-1 cell by LPS for 12 hours, the supernatant was taken after centrifugation to detect the content of TNF-α. Compared with the LPS group, the production of TNF-α from the THP-1 cells after incubation with GSPE through stimulation with LPS in LPS+GSPE group had obviously decreased (26.97 ± 0.72 vs. 58.07 ± 0.92 pg/ml, P < 0.01) (Fig. 7). Thus, GSPE can effectively inhibit the production of TNF-α in mononuclear cells and reduce the content of the inflammatory mediator TNF-α in rat plasma after MT, thereby playing an anti-inflammatory effect.

### GSPE attenuated trauma-induced THP-1 cell’s ROS overproduction

**Figure 8:**
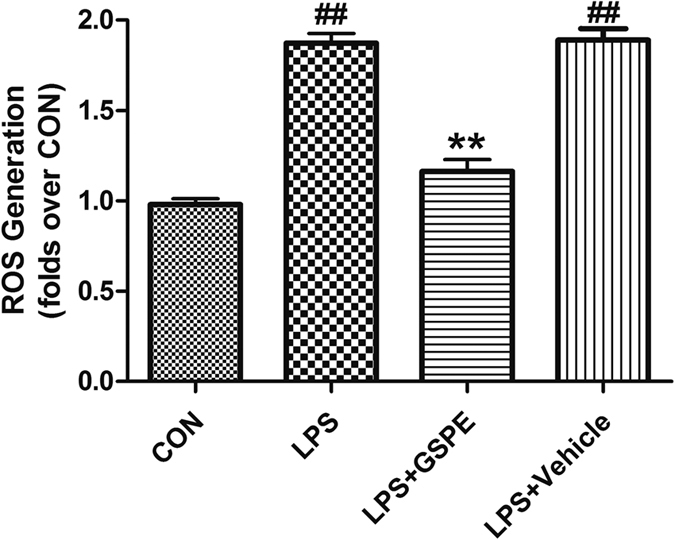
LPS Induced THP-1 cells’ ROS overproduction can be effectively attenuated by GSPE. The figure shows the intracellular ROS accumulation determined by using DCFH-DA in THP-1 cells. And the ROS overproduction induced by LPS was significantly attenuated by pretreatment with GSPE (100 μg/ml), ^##^P < 0.01 vs CON group, **P < 0.01 vs LPS group.

ROS can also activate monocyte macrophage, and enhance the expression of TNF-α gene, causing the abnormal increase of TNF-α concentration in plasma. The ROS content of THP-1 cells after incubation with GSPE through stimulation of LPS in the LPS+GSPE group was significantly lower than that in the LPS group (1.16 ± 0.04 vs. 1.87 ± 0.03, P < 0.01) (Fig. 8). Thus, GSPE can inhibit excessive oxidative stress reaction of myocardial cells and mononuclear cells stimulated by LPS in the microenvironment of inflammatory mediators, and effectively play its anti-inflammatory and antioxidant effects in the pathological process of MT leading to secondary cardiac insufficiency in rats.

### GSPE ameliorated TP-induced H9c2 cell calcium overload

**Figure 9:**
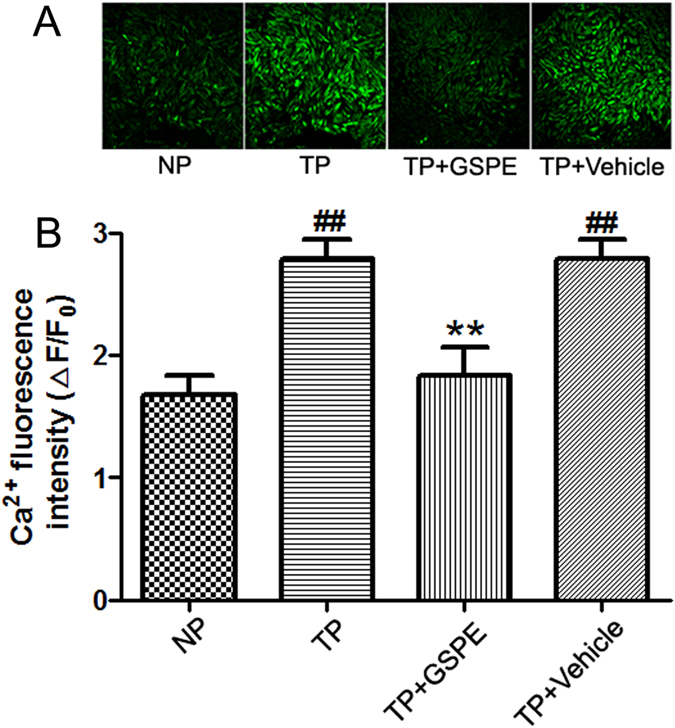
GSPE decreases myocardial Intracellular Ca^2+^ generation after trauma. (**A**) Representative images from confocal laser scanning microscope images of Ca^2+^ fluorescence in cells. (**B**) Ca^2+^ fluorescence intensity was indexed by ΔF/F_0_ (F_0_ represents the average fluorescence levels measured in the former 60 s, while F was the average fluorescence level in the later 180 s duration). n = 20~30 cells in each group. ^##^P < 0.01 vs CON group, **P < 0.01 vs Trauma group.

The fluorescence intensity of Ca^2+^ in a large number of H9c2 cells after the stimulation of TP in the trauma plasma group gradually increased compared with the normal group (2.80 ± 0.15 vs. 1.68 ± 0.16, P < 0.01). After drug interference, the number of myocardial cells in the trauma plasma groups was reduced (1.84 ± 0.23 vs. 2.80 ± 0.15, P < 0.01), with decreasing fluorescence intensity, close to normal group (1.84 ± 0.23 vs. 1.68 ± 0.16, P > 0.05). Thus, GSPE has an inhibitory effect on the abnormal rise of the content Ca^2+^ in myocardial cells caused by MT, i.e., calcium overload. (Fig. 9).

## Discussion

The application of GSPE as a powerful antioxidant in organ protection has attracted increasing attention^17^,^18^. Studies have shown that GSPE can effectively protect the body against hepatotoxicity, nephrotoxicity caused by a variety of drugs, chemicals^19^,^20^, and even improve beta-cell functionality under lipotoxic conditions^21^. GSPE can also play a neuroprotective effect by scavenging ROS to treat patients with ischemic stroke^22^. Moreover, after administration of GSPE, the heart of rat treated by ischemia/reperfusion showed significantly smaller infarct size than that in the control group, with significantly improved ventricular function^23^,^24^. Thus, this study aims to investigate whether GSPE can effectively alleviate myocardial cell apoptosis and improve heart function through its anti-inflammatory and antioxidative effects in the pathological process of MT leading to secondary cardiac insufficiency in rats.

Oxidative stress plays a critical role in the occurrence and development of ischemic heart disease, hypertension, arteriosclerosis, congestive heart failure and various cardiovascular diseases^25^,^26^,^27^. During the pathological process of secondary cardiac insufficiency caused by MT, myocardial superoxide anion and NO are produced massively, with rising ONOO^−^ formation and enhanced caspase-3 activity, thereby causing myocardial cell apoptosis, nuclear chromatin margination, mitochondrial swelling, dissolution and other pathological changes. Massive production of superoxide anion and NO is one of the important pathological bases of induced secondary cardiac insufficiency^8^. This experiment confirmed that after GSPE interference, the ROS content from H9c2 cells stimulated by TNF-α after MT was significantly lower than that in the trauma group. Thus, GSPE can effectively inhibit excessive ROS production from myocardial cells and block oxidative stress, which is a factor rink causing myocardial cell apoptosis.

The effect of Ca^2+^ in myocardial cell apoptosis has been widely recognized. Numerous evidence showed that, when myocardial cell is in the state of oxidative stress, a large number of Ca^2+^ from extracellular and intracellular organelles (i.e., mitochondria, endoplasmic reticulum) go into the cytoplasm, causing Ca^2+^ overload in myocardial cell and organelle damage, followed by apoptosis. Ca^2+^ overload is another important pathological basis of myocardial cell apoptosis under stress^28^,^29^. The results showed that the content of Ca^2+^ in H9c2 cells stimulated by plasma after MT continues to rise. In the GSPE interference group, given that the oxidative stress was inhibited, the degree of increase of the content of Ca^2+^ in H9c2 cells was significantly lower than that in the trauma group, and myocardial cell’s calcium overload was significantly improved.

**Figure 10:**
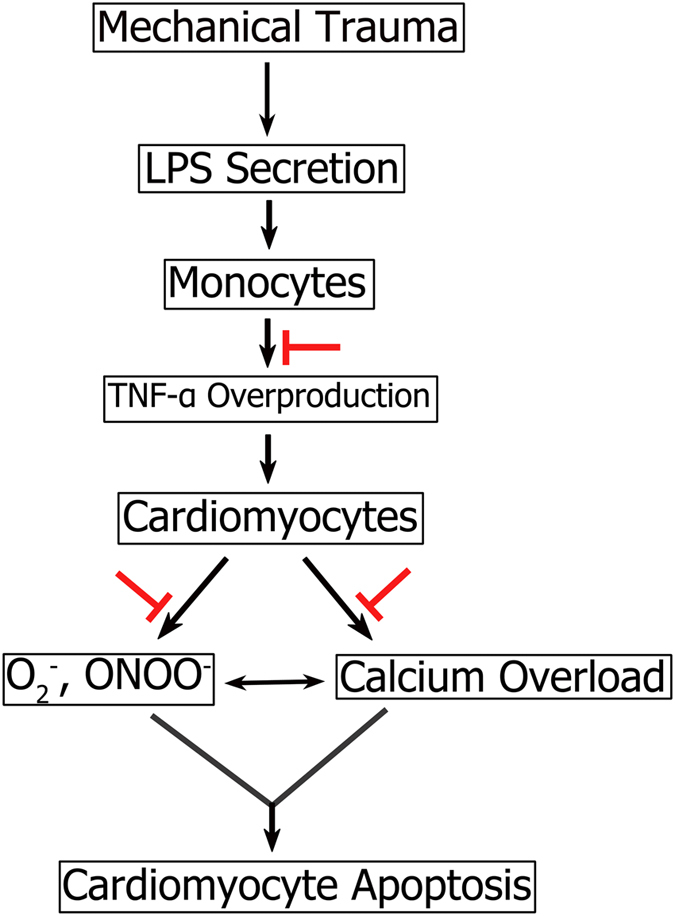
The mechanism of myocardial cell apoptosis caused by mechanical trauma. Patients with systemic mechanical trauma present intestinal barrier dysfunction, causing intestinal bacteria translocation and the release of toxins into the blood, resulting in a significant increase in the level of LPS in plasma. LPS, as a powerful inflammation factor, can activate monocytes, and enhance the expression of TNF-α gene, causing the abnormal increase of TNF-α. And increased TNF-α in plasma can induce overexpression of iNOS/NADPH oxidase, resulting in massive production of O_2_^−^ and superoxide anions in myocardial tissues. Consequently, oxidative stress reaction causes damage to the heart, resulting in secondary cardiac insufficiency after mechanical trauma. The current experiments showed that after GSPE interference, the content of TNF-α from monocytes stimulated by LPS had decreased significantly, as well as the content of inflammatory indicator TNF-α in plasma after mechanical trauma. Meanwhile, GSPE can also effectively inhibit excessive ROS production from myocardial cells and block oxidative stress. Furthermore, the degree of increased Ca^2+^ concentration in cardiomyocytes stimulated by plasma after mechanical trauma was significantly declined in GSPE interference group, and myocardial cell’s calcium overload was significantly mitigated (as shown in red arrows).

Patients with serious systemic MT present intestinal barrier dysfunction, causing intestinal bacteria translocation and the release of toxins into the blood, resulting in a significant increase in the level of LPS in plasma^2^. LPS, as a powerful inflammation factor, can activate TLR4 receptor in monocyte macrophage, start downstream NFκB and MAPK related signal pathway, and enhance the expression of TNF-α and IL-6 gene, causing the abnormal increase of TNF-α, IL-6 and other inflammatory indicators in plasma, and systemic inflammatory response syndrome, even MODS^30^,^31^,^32^,^33^. Our previous studies showed that abnormally increased TNF-α in plasma can induce overexpression of iNOS/NADPH oxidase, resulting in massive production of NO and superoxide anions in myocardial tissues and other parts of the body. Consequently, oxidative stress reaction causes damage to the heart, resulting in secondary cardiac insufficiency after MT^10^,^11^ (Fig. 10). Previous studies on GSPE mainly focused on the antioxidant effect in the aspect of organ protection. The current experiment showed that after GSPE interference, the content of TNF-α from THP-1 cells stimulated by LPS had decreased significantly, as well as the content of inflammatory indicator TNF-α in rat plasma after MT. Thus, GSPE can effectively inhibit the inflammatory indicator TNF-α in the pathological process of secondary myocardial cell apoptosis and cardiac insufficiency caused by MT to enhance heart protection from the upstream. The means of realizing this result need further investigation.

Intracellular Ca^2+^ is used as the second messenger during the processes of LPS-induced activation of TLR4 receptor in mononuclear macrophage and downstream NFκB and MAPK-related signaling pathways. The activation of the related calcium ion channels plays a critical role in regulating inflammatory indicators^33^. The TRP channel superfamily is a cation channel in the cell membrane that includes seven subfamilies. TRPM2 is the second member of the TRPM subfamily and is distributed in mononuclear cells, myocardial cells, and nerve cells etc. TRPM2 is a kind of transparent and non-selective cation channel of Ca^2+^ that plays an important role in oxidative stress reaction^34^,^35^. Wehrhahn J found that the TRPM2-mediated increase of Ca^2+^ in monomuclear cells is a key link of the LPS-induced activation of monomuclear cells and the release inflammatory factors. After downregulating the expression of the TRPM2 gene, the release of TNF-α, IL-6 and other inflammatory indicators from monomuclear cells have decreased^36^. The intracellular ADPR, NAD, cyclic ADP-ribose (cADPR), NADP, 2′-oxo-acetylated ADPR, and extracellular H_2_O_2_, TNF-α are known agonists of the TRPM2 channel^37^,^38^. The current experiment also showed that GSPE can also effectively inhibit excessive oxidative stress reaction produced from monomuclear cells stimulated by LPS. Further studies are needed to investigate whether GSPE can reduce H_2_O_2_-related and oxidative stress-related ADPR, NAD, cADPR and other agonists of the TRPM2 channel through scavenging oxygen free radicals, thus inhibiting the TRPM2-mediated increase of intracellular calcium ion and reducing the LPS-induced production of TNF-α.

As a causative role in cardiomyocyte apoptosis after mechanical trauma in rats, TNF-α level peaked at 1.5 h after trauma^10^. And in order to guarantee GSPE’s peak level cover the most important period of TNF-α massive production in rats. GSPE was administered 30 minutes before trauma. This treatment may limit the clinical application as it is impossible to treat patients before they were traumatized. Thereafter, further studies are needed to investigate the effects of GSPE given after trauma.

In summary, GSPE has effective anti-inflammatory and antioxidant effects in the pathological process of secondary myocardial cell apoptosis and cardiac insufficiency caused by MT, thereby easing myocardial damage and improving cardiac function. GSPE also has cardio-protective effects. This study provides a theoretical basis for developing GSPE as a drug for preventing secondary myocardial cell apoptosis and cardiac insufficiency caused by MT and MODS secondary to trauma.

## Methods

### Materials

Proanthocyanidin was extracted from grape seeds, purity is more than 99%, which contains approximately 57% dimeric, 14% trimeric, and 9% tetrameric oligomeric proanthocyanidins, and a small amount of high-molecular-weight oligomeric procyanidins, purchased from Tianjin Peak Natural Product Research Development Co., Ltd. (Tianjin, China). DMEM was purchased from Gibco BRL Co., Ltd. (Grand Island, NY, USA). H9c2 cells were obtained from American Type Culture Collection (ATCC, Manassas, VA, USA; CRL-1446). THP-1 cells were obtained from China Infrastructure of Cell Line Resources. The DCFH-DA ROS Detection Kit was purchased from Beyotime Institute of Biotechnology (Nanjing, China). The TUNEL (terminal deoxynucleotidyl transferase-mediated dUTP nick-end labelling) apoptosis detection kit was purchased from Roche (Shanghai, China). 3-(4,5-Dimethyl-2-thiazolyl)-2,5-diphenyl-2-H-tetrazolium bromide (MTT), RPMI-1640 media, 10% heat-inactivated foetal bovine serum (FBS), Fluo-4/Am were purchased from Sigma (USA). The TNF-α assay kit was purchased from Shenggong (Shanghai, China). The modified Noble-Collip drum was manufactured by Dalian University of Technology (Dalian, China)^8^. The laser confocal microscope was purchased from Leica Co. (USA). And the electron microscope was JEM-2000EX (Japan). BL-420 biological experimental system and pressure sensors were purchased from Taimeng Sciences and Technology Limited (Chengdu, China). The BX51 fluorescence microscope was purchased from Olympus Co. (Japan). The microplate reader was purchased from BioTek (VT, USA).

### Ethics statement

The experiments were performed in accordance with the Guide for the Care and Use of Laboratory Animals published by the US National Institutes of Health and the Guide for the Care and Use of Laboratory Animals’ protocol published by the Ministry of the People’s Republic of China (issued 3 June, 2004), and were approved by Dalian Medical University Committee on Animal Care. All surgery was performed under xylazine and ketamine anesthesia, and all efforts were made to minimize suffering.

### Induction of nonlethal mechanical trauma in Rats

The animals were housed in a room maintained at a constant temperature of 20 ± 2 °C and under a 12:12-h light-dark cycle at 60–80% humidity, with food and water available ad libitum. All surgery was performed under anaesthesia with a 1:1 mixture of xylazine (10 mg/kg) and ketamine (70 mg/kg) given by the intraperitoneal route, and all efforts were made to minimize suffering. Male Sprague Dawley rats (210 ± 20 g) were anesthetized with the mixture of xylazine and ketamine, and were placed in a Noble-Collip drum (200 revolutions at a rate of 35 rpm) to induce a nonlethal mechanical trauma as we previously described^8^,^10^,^39^. The rats were normally active in their cages about 2 hours post-trauma. The 12-hour survival rate is 95%. The rats were randomized divided into Control group, Trauma group, Trauma+ GSPE group, and Trauma+ Vehicle group. The Trauma+ GSPE group and Vehicle group receive one of the following solutions intraperitoneally 30 minutes before trauma: Vehicle group (0.9% NaCl, 4 ml/kg); Trauma+ GSPE group (Proanthocyanidin extracted from grape seeds, 40 mg/kg)^40^,^41^. Control rats were subjected to the same revolution but taped on the inner shelf of the drum to avoid the injury. 12 hours after trauma, a polyethylene catheter (PE-50) was inserted into rats’ left ventricular cavity through the right carotid artery, and left ventricular developmental pressure (LVDP), the maximal positive and negative values of the instantaneous derivative of left ventricular pressure (+dP/dtmax and −dP/dtmax) were measured by BL-420S Biological Signal Analytical System^39^.

### Determination of Myocardial Apoptosis

Myocardial apoptosis (12 hours after trauma) was determined by TUNEL (terminal deoxynucleotidyl transferase dUTP nick end labeling) staining and caspase-3 activity assay. Apoptotic cells in left ventricular tissues were detected with TUNEL labeling using an *In Situ* Cell Death Detection Kit, Fluorescein, following the manufacturer’s instructions. After TUNEL labeling, nuclei were stained with DAPI, and the TUNEL positive cells were observed using a microscope. Total nuclei (DAPI staining, blue) and the TUNEL positive nuclei (green) in each field were determined in five randomly chosen fields. The caspase-3 activity was determined by measuring the generation of the fluorogenic cleavage product methylcoumarylamide (AMC) from the fluorogenic substrate Ac-DEVD-AMC at 360 nm excitation wavelength and 460 nm emission wavelength. AMC standards were used to quantify activity levels. Specificity of the assay was confirmed by addition of the specific caspase-3 inhibitor Ac-DEVD-CHO to the reaction mixture.

### Most appropriate concentration of GSPE detected by MTT

A 100 μl H9c2 cell suspension was loaded into each well of a 96-well plate and was cultured in the logarithmic growth phase until the cell concentration reached 5 × 10^7^/L. The cells were then treated with GSPE at a series of diluted concentrations (60, 80, 100, 120, or 140 μg/ml, respectively), except the cells in the blank group (without vehicle) and control group (with vehicle) for 24 hours. Before harvesting, the cells were incubated with 20 μl of MTT in medium for 4 hours at 37 °C. The viable cells converted the MTT to a blue-purple colour after dissolving in 150 μl of dimethyl sulfoxide (DMSO). The absorbance at 570 nm was measured using a microplate reader. All experiments were carried out in triplicate. Cell viability (%) = [(absorbance of cells treated with GSPE)/absorbance of control cells] × 100%^42^,^43^.

### Determination of TNF-α production and intracellular ROS accumulation

On the one hand, the serum of 4 different groups’ rats was extracted from aortic artery 1.5 hours after trauma^10^. On the other hand, THP-1 cells^44^ were stimulated by LPS as inflammatory model and the THP-1 cells were randomly divided into 1) Control group (incubated with PBS for 12 hours); 2) LPS group (incubated with 1 μg/ml LPS for 12 hours)^45^; 3) LPS + GSPE group (0.5 hours pretreated with 100 μg/ml GSPE and incubated with 1 μg/ml LPS for 12 hours)^46^; 4) Vehicle group (0.5 hours pretreated with NS and incubated with 1 ug/ml LPS for 12 hours). The cells were then centrifugated and the supernatant was collected for TNF-α measurement. And then the TNF-α concentration of above samples were measured using enzyme-linked immunosorbent assay kits.

The intracellular ROS accumulation in H9c2 (China Infrastructure of Cell Line Resources)^47^ or THP-1 cells was measured by 2′, 7′-dichlorodihydro fluorescin diacetate (DCFH-DA) using an intracellular ROS assay kit. H9c2 or THP-1 cells cells were pre-loaded with DCFH-DA by exposure to freshly prepared 100 μM DCFH-DA in the culture medium for 30 minutes at 37 °C. On the one hand, the DCFH-DA loaded H9c2 cells were treated with 50% trauma serum (the serum from rats after nonlethal mechanical trauma) alone or trauma serum combined with GSPE/NS for 3 hours; On the other hand, the DCFH-DA loaded THP-1 cells were treated with 1 ug/ml LPS alone for 12 hours or 1 ug/ml LPS combined with pretreating with 100 mg/ml GSPE/NS for 0.5 hours. Then the DCFH fluorescence of the cell was measured with excitation and emission settings of 485 nm and 530 nm by a microplate reader.

### Measurements of Ca^2+^ transients in cardiomyocytes

Intracellular Ca^2+^ levels were monitored using the Ca^2+^-sensitive fluorescent indicator, Fluo-4AM. Cultured H9c2 cell on glass coverslips were loaded with Fluo-4AM (5 μmol/L) in Tyrode’s solution at room temperature for 30 min, the dish was washed with Tyrode’s solution with 1% BSA to remove any non-specific staining outside the cell. And were randomly incubated with one of the following agents at 37 °C for 30 min: 1) Control group (200 μl, PBS); 2) Trauma group (200 μl, PBS); 3) GSPE group (100 mg/ml, 200 μl, GSPE)^46^; 4) Vehicle group (200 μl, NS); Then the cell fluorescence intensity was examined using a confocal laser scanning microscope. A 494 nm excitation wavelength provided by an argon laser was used to illuminate Fluo-4, and fluorescence was detected at emission wavelength 510 nm and above. 60 s later, we add 20 μl 50% normal serum (the serum from the control rats) into control group, and 20 μl 50% trauma serum into the other group Changes in Ca^2+^ levels were represented by relative fluorescence intensity (F/F_0_%), where F_0_ represents the average fluorescence levels measured in the former 60 s, while F was the average fluorescence level in the later 180 s duration^48^,^49^.

### Electron microscope analysis of cardiomyocytes

12 hours after trauma, samples of the left ventricle myocardium (in the region of the heart apex) were cut into pieces less than 1 mm^3^ and were fixated with 2.5% glutaraldehyde on 0.1 M phosphate buffer (pH 7.2-7.4) for 24 hours, then fixated once more by 1% OsO_4_ solution, contrasted by 2% uranyl acetate solution on 70% ethanol, dehydrated by the generally accepted method and viewed through JEM-2000EX electron microscopes^50^.

### Statistical analysis

Statistical comparisons between groups were performed by the use of t-test or one-way analysis of variance (ANOVA) test and Tukey test. Data were presented as mean ± sem. (GraphPad Software, USA) P values less than 0.05 were considered to be statistically significant.

## Additional Information

**How to cite this article:** Ma, S. *et al*. Mitigation Effect of Proanthocyanidin on Secondary Heart Injury in Rats Caused by Mechanical Trauma. *Sci. Rep.* **7**, 44623; doi: 10.1038/srep44623 (2017).

**Publisher's note:** Springer Nature remains neutral with regard to jurisdictional claims in published maps and institutional affiliations.
